# Acupressure and Ginger to Relieve Nausea and Vomiting in Pregnancy: a Randomized Study

**DOI:** 10.5812/ircmj.12984

**Published:** 2013-09-05

**Authors:** Farzaneh Saberi, Zohreh Sadat, Masoumeh Abedzadeh-Kalahroudi, Mahboobeh Taebi

**Affiliations:** 1 Department of Midwifery, Faculty of Nursing and Midwifery, Kashan University of Medical Sciences, Kashan, IR Iran; 2 Trauma Nursing Research Center, Kashan University of Medical Sciences, Kashan, IR Iran; 3 Trauma Research Center, Kashan University of Medical Sciences, Kashan, IR Iran; 4 Department of Midwifery, Faculty of Nursing and Midwifery, Isfahan University of Medical Sciences, Isfahan, IR Iran

**Keywords:** Ginger, Acupressure, Nausea, Vomiting, Pregnancy

## Abstract

**Background:**

Nausea and vomiting of pregnancy (NVP) is the most common medical condition of pregnancy, affecting up to 85% of expecting mothers. NVP can have serious adverse effects on the quality of a woman's life, social, and domestic functioning, and her general well-being. Therefore, it is very important to treat this condition.

**Objectives:**

The effectiveness of ginger and acupressure in the treatment of NVP was compared in the present study.

**Patients and Methods:**

159 eligible pregnant women with symptoms of mild to moderate nausea and/or vomiting before 16 weeks gestational age participated in a 7-day clinical trial. They were divided randomly into three groups: the acupressure, ginger, and control. Participants did not receive any intervention for three days and interventions were performed for the women in acupressure and ginger groups for four days. No intervention was performed for the control group. Data was collected by self-recorded symptoms according to the Rhodes index. Data was analyzed by ANOVA, Kruskal-Wallis, Chi-square, and Fisher exact tests for quantitative and qualitative variables.

**Results:**

There were no statistical differences in the baseline demographics between the three groups. ANOVA test showed that there were significantly differences in mean difference Rhodes index scores (vomiting, nausea, retching and total score) in the three groups (P < 0.001).

**Conclusions:**

Ginger is more effective than acupressure to relieve mild to moderate nausea and vomiting in symptomatic pregnant women in less than 16 weeks of gestational age.

## 1. Background

Nausea and vomiting of pregnancy (NVP) is the most common complication of pregnancy which occurs up to 85% of mothers (1). This problem starts about the 4th week of pregnancy, and usually continues to the 16th week in a few mothers (2). The etiology of NVP is unknown. It occurs due to hormonal, immunological, and anatomical changes, although in many studies were not accepted (3). NVP develops to hyperemesis gravidarum in less than 2% of women. This complication characterized by repeated vomiting leading to fluid and electrolyte imbalance, nutrition deficiency, and a weight loss of more than 5% of the prepregnancy weight, often leading to hospitalization (4). Women with hyperemesis in first pregnancy have a high risk for recurrence in next pregnancy (5). Studies in Iran showed that the frequency of severe nausea and vomiting was 16% to 21.7% (6, 7). Severe NVP may lead to depression, feelings of inadequacy, loss of working hours, hospitalization and termination of pregnancy (8, 9). NVP has adverse effects on the quality of a woman's life, social, relationship with family, and her general health; then, properly and effectively treatment is very important in this condition (3, 10). It is typically treated with pharmacologic and nonpharmacologic (acupressure, acustimulation, acupuncture, ginger and vitamin B6) antiemetic (11, 12). Several researches have been performed about the effect of ginger or acupressure on nausea and vomiting in pregnancy in Iran (13-16). In addition, surveys have shown that some herbal products were recommended to treat NVP by midwives (17). Ginger is an herb which its rhizome is used as spice and medicine. It can be used fresh, dried and powdered, or as a juice or oil. It is commonly used to treat various types of stomach problems (18). Since years ago, ginger has been used for treating nausea and vomiting in early pregnancy (13, 19). Studies have used powder or capsule forms to relieve NVP in Iran (13, 14, 20).

Heitmann et al. in a review study reported that the risk of congenital malformations, stillbirth / perinatal death, preterm birth, low birth weight, or low Apgar score did not increase when ginger was used during pregnancy (21). Therefore, a safe and effective treatment choice for NVP is ginger (22). The use of ginger products may be helpful to relieve nausea and vomiting, but the evidence of effectiveness was limited (23).

The effectiveness of acupressure at P6 point compared to the placebo group in reducing NVP has been reported in various trials (24). Acupressure works on the precardium 6 (P6 or Neiguan) as acupressure point on the wrist. This point is found by measuring, with the mother´s own finger, three fingers width up from the inner wrist crease where the hand joins the arm, approximately where the buckle of watchstrap might rest (25). A group of evidence-based medicine reviewers, reviewed the use of P6 for nausea and vomiting, and resulted that it is an effective method for reliving postoperative nausea. They concluded that acupressure may be a useful method for the management of nausea and vomiting in a variety of patients, but accurate trials are needed (26).

## 2. Objectives

To our knowledge, the use of ginger and acupressure (two nonpharmacological therapies) has not been compared in a randomized clinical trial. Therefore, comparison of the effectiveness of ginger and acupressure in the treatment of nausea and vomiting in pregnancy was the aim of our study.

## 3. Materials and Methods

The research ethics committee of the Kashan University of Medical Sciences approved the study with the number code of 29/5/1/4406 in 06/11/2007. It was registered in the Iranian registry of clinical trial with this number: 201103192699N4. This randomized control clinical trial was performed from 10 November 2008 to 20 September 2009 in antenatal clinic at Naghvi hospital, Kashan, Iran. Inclusion criteria were: (1) willingness to participate in the study, (2) having mild to moderate nausea and/or vomiting, (3) less than 16 weeks’ gestation, (4) singleton pregnancy, (5) literate, (6) no history of other diseases such as gastrointestinal disorder, (7) not using other methods for treatment of NVP in the past 3 weeks, (8) able to eat the ginger capsules or place the wristbands as prescribed in the correct placement, and (9) lived in Kashan.

Women were excluded if they were unable to return for a follow-up visit one week later, had complications when using ginger or wristbands, the advised method for treatment failed to relieve nausea and vomiting, and NVP was progressing to severe (> 5 episodes per day).

After obtaining verbal informed consent, women underwent general physical examinations and routine obstetric evaluations. They were subsequently randomized into three groups (ginger, acupressure and control) using a table of random numbers.

At first, the demographic form including age, age of marriage, gestational age, occupation, parity, wanted or unwanted pregnancy and education was completed. Women were instructed not to take any other medications except the treatment advised by the researchers. Women were followed for 7 days. They did not receive any intervention for the first three days but interventions were performed for the acupressure and ginger groups for the next four days. All women in the three groups were instructed to go on diet during the study [split their meals into frequent small ones, rich in carbohydrates and low fat. Also avoiding or not to eat food that may actually make nausea worse, try eating before or as soon as you feel hungry, stop smoking, eat dry bread or cookie on awaking, avoiding fried, odorous, spicy, greasy, or gas forming foods, maintaining good posture, drinking cold, clear, and carbonated or sour fluids (27)].

The benefits, risks and effectiveness of new intervention were described. We explained that the privacy of women and their personal information would be protected. In addition, at the end of the study, the women would be informed about the results. They were asked to start a medication if the advised treatment failed or vomiting was more than 5 times per day and excluded the study.

All eligible women received a package containing 14 copies of Rhodes index of nausea and vomiting. In addition, we instructed to evaluate their symptoms every 12 hours (twice daily for seven consecutive days). At a 7-day follow-up, women reported the severity of their symptoms by the Rhodes index form. The Rhodes index was expanded to eight items. Eight 5-point self-report items measure the patient's perception of duration of nausea, frequency of nausea, distress from nausea, frequency of vomiting, amount of vomiting, distress from vomiting, frequency of retching, and distress from retching. This form arranges the eight items, which describes the level of symptoms. The likert- type scale for each item was scored from zero (indicating minimal or no symptom) to four (representing the worst symptom). The item scores were summed for a total score with a range of 0 to 32. Patients were asked to evaluate the syndrome every 12 hours on a 5-point scale (28). The Rhodes index has been used for assessment of NVP in some studies in Iran (7, 29-33), and other countries (24, 34, 35). In Iranian research, its validity was confirmed by content validity, and its reliability was calculated and confirmed by Cronbach's alpha (α = 0.898) (29). Also its coefficient correlation was high in other researches (with Cronbach alphas of 0.77 in the United Kingdom, 0.897 in the USA, and 0.929 in China) (36-38).

Each women in acupressure group was given a pair of sea band (acupressure wristband) (Sea- Band, the U.K., Ltd., Leicester, England) and trained to use it continuously (remove only when bathing) for four days (From the fourth to seventh day) in the appropriate place in both hands. Sea band is a buttoned elastic wristband which is used to pressure on the Neiguan point.

**Figure 1.**
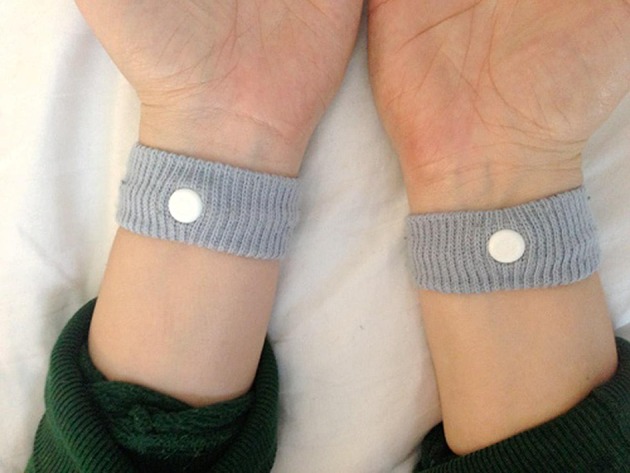
Location of Acupressure Wristband

Each woman in ginger group received 12 ginger capsules 250 mg (with the brand named Zintoma made in Goldaroo manufacturing Pharmaceutical Company) for 4 days (during the four to seven days) and daily 3 capsules. No intervention was performed for the control group during 7 days.

The women were called twice: once in the fourth and another in the eighth day. On the fourth day, we answered the women's questions in three groups; also, we reminded the use of ginger capsules and wristbands in the intervention groups. On the eighth day, we thanked the women for their participation in this study and requested to hand over the Rhodes forms for evaluation of their responses to the advised methods of treatment. NVP was evaluated by the Rhodes index score.

Data was analyzed by SPSS software version 14. In the descriptive analysis were represented as means and standard deviation, while the categorical variables were represented as frequency and percentages. ANOVA, Kruskal-Wallis, Chi-square and Fisher exact tests were used for quantitative and qualitative variables.

The hypothesis tested whether ginger and acupressure were different in reduce nausea, vomiting and retching symptoms. These were indicated by mean difference Rhodes index scores between the three groups by ANOVA test. It was calculated by mean Rhodes index scores in four days after the intervention (post intervention) minus in three days before the intervention (pre intervention).

A pilot study was performed and sample size was calculated (n = 10). The mean differences Rhodes index scores were 4.2 and 7.5 in acupressure and ginger groups, respectively. To reject the null hypothesis of improvement in symptoms with a power of 80% and a significance level of 5%, sample size of 48 women per group was calculated. Considering 10% loss in follow up, 53 women in any group were needed. A significance level of P < 0.05 was used for all tests.

## 4. Results

**Figure 2.**
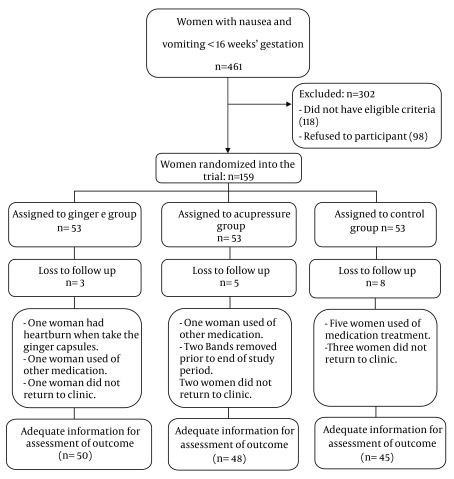
Trial Profile of Recruitment and Randomization to Acupressure, Ginger or Control Groups

All women were included in the intention to treat (ITT) analysis. From 10 November 2008 to 20 September 2009, 461 pregnant women were screened, and among them, 159 women were recruited and 302 were excluded. In ginger group, one woman had heartburn when taking the ginger capsules, one woman used medication, and one woman did not return to clinic. In acupressure group, one woman used other medication, two removed their bands prior to the end of study period, and two women did not return to clinic. In control group, five women used medication treatment, and three women did not return to the clinic. They were excluded from the study. Finally, there were 50 women in ginger groups, 48 in acupressure and 45 in control. Analyses were performed on 143 women (Figure 2). 

We checked normal distribution in variables. All variables had normal distribution apart from age and age of marriage. ANOVA test was used to compare variables with normal distribution and Kruskal-Wallis test was used to compare non-normal variables in three groups. There were no statistically significant differences in the baseline characteristics between the three groups (Table 1). 

**Table 1.** Baseline Characteristics of the Patients^a^

Characteristics	Acupressure (n = 53)	ginger (n = 53)	control (n = 53)	P-value
**Age (years)**	25.68 ± 4.64	26.64 ± 6.18	25.79 ± 3.64	NS^b^(0.531)
**Age of marriage **	19.75 ± 1.96	18.66 ± 4.41	19.39 ± 1.93	NS^b^(0.063)
**Gestational age (weeks) **	9.32 ± 2.38	8.78 ± 2.32	9.11 ± 0.18	NS^c^(0.441)
**Occupation**				NS^d^(0.479)
Housewife	51 (96.2)	50 (94.3)	48 (90.6)	
Employee	2 (3.8)	3 (5.7)	5 (9.4)	
**Parity**				NS^e^(0.187)
Nulliparous	33 (62.3)	26 (49.1)	24 (45.3)	
Multiparous	20 (37.7)	27 (50.9)	29 (54.7)	
**Pregnancy**				NS^e^(0.288)
wanted	50 (94.3)	45 (84.9)	47 (88.7)	
Unwanted	3 (5.7)	8 (15.1)	6 (11.3)	
**Education**				NS^e^(0.557)
Less than high school	20 (37.7)	15 (28.3)	19 (35.8)	
High school or more	33 (62.3)	38 (71.7)	34 (64.2)	

^a^ Data are presented as mean ± SD or No., (%)

^b^ Kruskal-Wallis test was used

^c^ ANOVA test was used

^d^ Fisher exact test was used

^e^ Chi-squared test was used

**Figure 3.**
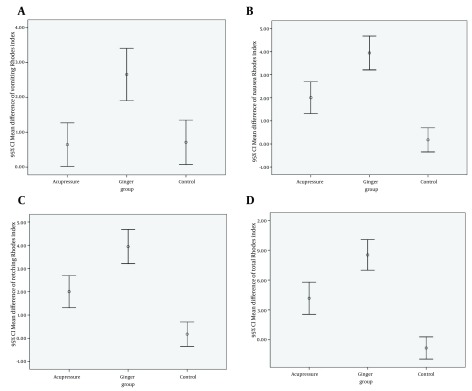
Comparing the Mean Difference Rhodes Index Scores of Vomiting (A), Nausea (B), Retching (C), and Total (D) between the Three Groups

We compared the mean difference Rhodes index scores between the three groups. The mean difference Rhodes index scores calculated by mean Rhodes index scores in pre intervention (three days before intervention) minus mean Rhodes index scores in post intervention (four days after intervention). It was significantly greater in ginger group than acupressure and control groups. ANOVA test showed that there were significant differences in the mean differences in vomiting, nausea, retching and total scores between the three groups (P<0.001). These results were presented by error bar plot in Figure 3. 

Paired t-test was also used to compare the mean pre and post intervention scores. Results indicated that there were significant differences in the mean pre and post intervention in ginger and acupressure groups. No significant differences were found apart from vomiting in control group (Table 2). 

**Table 2.** The Mean Pre and Post Intervention and Difference Rhodes Index Scores in the Three Groups^a^

Groups Variables	Ginger (n = 50)				Acupressure (n = 48)				Control (n = 45)				ANOVA
	Pre intervention	Post intervention	Paired t test	difference	Pre intervention	Post intervention	Paired t test	difference	The first 3 days	The second 4 days	Paired t test	difference	P value
**Vomiting**	5.14 ± 3.10	2.49 ± 2.24	0.00	2.66 ± 2.64	5.14 ± 3.00	4.49 ± 2.76	0.043	0.64 ± 2.14	5.14 ± 2.13	4.50 ± 2.76	0.029	-0.71 ± 2.12	< 0.001
**Nausea**	8.42 ± 2.25	4.48 ± 2.06	0.00	3.94 ± 2.58	9.22 ± 2.31	7.21 ± 2.91	0.00	2.00 ± 2.37	8.41 ± 2.21	8.24 ± 2.53	0.50	0.18 ± 1.74	< 0.001
**Retching**	4.34 ± 2.13	2.33 ± 1.63	0.00	2.01 ± 1.56	4.35 ± 2.29	2.82 ± 2.03	0.00	1.52 ± 1.86	4.34 ± 2.19	4.65 ± 2.01	0.137	0.31 ± 1.36	< 0.001
**Total score**	17.91 ± 6.11	9.30 ± 4.68	0.00	8.61 ± 5.24	17.91 ± 5.90	13.74 ± 6.66	0.00	4.17 ± 5.53	17.90 ± 5.30	18.75 ± 5.60	0.137	-0.84 ± 3.72	< 0.001

^a^ ANOVA were used to compare the mean difference scores

Tukey post hoc test was performed and the results showed that the mean differences in vomiting, nausea, retching, and total scores between the groups were significantly different except for vomiting score between acupressure and control groups (P = 0.98), and retching score between acupressure and ginger groups (P = 0.29).

One-way repeated measure ANOVA was used to compare three group means of vomiting, nausea, retching and total score in the first to seventh days. These means were statically significant between the three groups (Table 3). 

**Table 3.** Mean and Standard Deviation of Vomiting, Nausea, Retching and Total Rhodes Index Score in Days 1 to 7 in the Three Groups

Variables and Groups	The First Day	The Second Day	The Third Day	The Fourth Day	The Fifth Day	The Sixth Day	The Seventh Day	P
**Vomiting**								< 0.001
Ginger	4.62 ± 4.05	5.24 ± 3.47	5.58 ± 3.61	3.06 ± 3.07	2.42 ± 2.73	1.90 ± 2.32	2.58 ± 3.19	
Acupressure	5.42 ± 3.92	5.54 ± 3.40	4.46 ± 3.15	4.90 ± 3.42	4.69 ± 3.55	4.15 ± 3.29	4.25 ± 3.38	
Control	5.23 ± 2.53	5.08 ± 2.94	5.10 ± 2.82	5.37 ± 2.91	6.26 ± 3.42	6.12 ± 3.40	5.66 ± 3.10	
**Nausea**								< 0.001
Ginger	8.92 ± 2.44	7.90 ± 2.88	8.46 ± 2.55	5.88 ± 2.17	4.44 ± 2.26	4.00 ± 3.02	3.62 ± 3.15	
Acupressure	10.18 ± 2.19	8.93 ± 3.17	8.55 ± 2.61	7.32 ± 3.60	6.83 ± 3.70	6.68 ± 2.71	8.03 ± 4.11	
Control	9.17 ± 2.39	7.90 ± 3.28	8.17 ± 2.80	7.82 ± 3.68	8.71 ± 3.35	9.35 ± 3.03	7.08 ± 3.0	
**Retching**								< 0.001
Ginger	4.24 ± 2.56	4.32 ± 2.08	4.42 ± 2.22	3.10 ± 1.51	2.34 ± 1.33	2.02 ± 1.98	2.12 ± 2.27	
Acupressure	5.06 ± 2.81	5.12 ± 2.42	4.56 ± 1.89	3.56 ± 2.48	3.37 ± 2.04	3.12 ± 2.42	3.66 ± 2.47	
Control	4.85 ± 2.51	3.79 ± 2.51	4.39 ± 2.70	4.61 ± 2.26	4.70 ± 2.01	4.92 ± 2.70	4.48 ± 2.25	
**Total**								< 0.001
Ginger	17.78 ± 7.43	17.46 ± 7.67	18.46 ± 5.98	12.04 ± 5.38	9.20 ± 5.24	7.92 ± 5.40	8.32 ± 7.48	
Acupressure	19.27 ± 7.17	18.20 ± 6.72	16.19 ± 5.51	14.39 ± 8.53	13.50 ± 8.12	12.56 ± 7.26	14.56 ± 8.66	
Control	19.25 ± 5.16	16.78 ± 6.95	17.67 ± 7.21	17.81 ± 7.50	19.67 ± 6.70	20.41 ± 7.58	17.23 ± 6.91	

Analysis showed that total Rhodes index scores reduced 49% in ginger group and 29% in acupressure group. It was raised up to 0.06% in control group (Table 4). 

**Table 4.** The Reduction Percentage of Rhodes Index Scores in the Studied Groups

Variable	Ginger (n = 50)	Acupressure (n = 48)	Control (n = 45)
**Vomiting**	52%	19%	-0.24%
**Nausea**	48%	29%	0.03%
**Retching**	46%	37%	-0.09%
**Total Score**	49%	29%	-0.06%

In general, the women in ginger group found that using this method was useful for relieving nausea, vomiting, and retching in pregnancy.

## 5. Discussion

As our knowledge, this is the first randomized, prospective trial to compare the effectiveness of ginger and acupressure in the treatment of NVP in referring women to antenatal clinic. Many studies had been performed and provided the information of the treatment of NVP, both pharmacological and nonpharmacological methods.

Results in this trial showed that ginger was effective in treating nausea, vomiting and retching. In Ozgoli et al. study, the experimental group received 250 mg capsules of ginger, 4 times a day for 4 days, and the control group took placebo with the similar prescription form. They found that ginger was an effective herbal therapy for relieving nausea and vomiting, and an improvement in nausea symptoms during pregnancy was reported by the most of pregnant women in the ginger group (13). In a randomized controlled study, intervention group took 1 g/day for 4 days. In that trial, an improvement in nausea symptoms was reported by 82.8% of women in the ginger group (20). However, the results in the present study showed that vomiting and nausea in the ginger group decreased 52% and 48% respectively. This may take place due to the lower ginger dose in our study (750 mg/day versus 1 g/day).

In addition, randomized studies results have shown statistically significant effects of acupressure in the treatment of nausea, vomiting, and retching symptoms (P < 0.001) (24, 39). In our study, acupressure was effective in relieving nausea, vomiting and retching. This finding is consistent with the results of jamingorn study; however, is inconsistent with the findings of Sinha et al. study. In their trial, the frequency of nausea and vomiting during labor and delivery did not reduce when the acupressure wristbands were applied bilaterally on women hands (40). It is possible because there was no control group in their study and two groups revived intervention, one group in the P6 point and the other one in the sham point. Then, there was a placebo effect in both the intervention and placebo groups.

In previous studies the efficacy of ginger and vitamin B6 was compared with placebo for the treatment of nausea and vomiting in pregnancy. These studies reported that ginger and vitamin B6 were useful for the management of nausea and vomiting in pregnancy, and ginger was more effective than vitamin B6 (20, 41). Also in a randomized study, the effectiveness of acupressure and vitamin B6 in relieving symptoms of nausea and vomiting in early pregnancy has been compared. Finding showed statistically significant decrease in nausea, retching, and vomiting symptoms in both acupressure (P < 0.001) and vitamin B6 groups (P < 0.001) (24).

To our knowledge, this is the first randomized controlled trial study which compared the effectiveness of ginger and acupressure in alleviating mild to moderate nausea, vomiting and retching in pregnancy. According to Table 2 the mean difference Rhodes index scores in ginger group was more than acupressure and control groups. There were statistically significant differences in the mean difference Rhodes index scores (vomiting, nausea, retching and total) between the three groups (P = 0.00). 

Total reduction percentage of Rhodes Index scores in the present study were 49%, 29% and -0.06% in ginger, acupressure and control groups respectively. These findings indicated that ginger is more effective than acupressure in treating nausea and vomiting.

In conclusion, ginger is more effective than acupressure in alleviating mild to moderate NVP in symptomatic women before 16 weeks’ gestation of pregnancy. Since this study was performed on mild to moderate nausea and vomiting, the results could not be generalized to severe nausea and vomiting, And it can be considered as a limitation. We suggest comparing the effectiveness of ginger and acupressure to relieve severe nausea and vomiting in pregnancy in future clinical trials.
